# Clinical Use of Lung Ultrasound by Emergency and Intensive Care Physicians: A Swiss National Survey

**DOI:** 10.1002/jcu.70025

**Published:** 2025-07-30

**Authors:** Norah Villars, Thomas Berlet, Luca Cioccari

**Affiliations:** ^1^ Department of Intensive Care Medicine Kantonsspital Aarau Aarau Switzerland; ^2^ Department of Intensive Care Medicine University of Bern, Bern University Hospital Bern Switzerland

**Keywords:** critical care, emergency medicine, lung, survey, ultrasonography

## Abstract

**Purpose:**

Lung ultrasound (LUS) has gained popularity in the emergency department (ED) and intensive care unit (ICU). However, little is known about its use, training, indications, and implementation barriers. Therefore, we performed a survey to evaluate the current practice of LUS among ED and ICU physicians with varying experience levels across regions in Switzerland.

**Methods:**

A 27‐question online survey was disseminated across 108 EDs and 75 ICUs using snowball sampling via department heads.

**Results:**

Of all 198 participants from 183 invited departments (49.0% ED physicians, 42.3% ICU physicians, 8.7% from other specialties), 190 (95.5%) use ultrasound and 164 (82.8%) use LUS in their clinical practice. Predominantly, LUS is utilized for evaluating dyspnea (95.9%), shock (76.2%), and hypoxemia (73.5%). ICU physicians used LUS more for invasive procedures, while less experienced physicians had lower certification rates. Standardized protocols for LUS examination or documentation were reported by only 22.5% and 38.9% of responders, respectively. The main barriers identified were time constraints, lack of training opportunities, and underestimation of LUSs diagnostic value.

**Conclusions:**

LUS is widely adopted and considered highly relevant by ED and ICU physicians. However, disparities in usage and proficiency were observed between ICU and non‐ICU physicians, as well as between more and less experienced practitioners. Addressing the identified training gaps and promoting standardized protocol adoption are imperative for optimizing LUSs integration into patient care.

## Graphical abstract

Lung ultrasound is widely used by emergency and ICU physicians, especially for evaluating dyspnea, shock, and hypoxemia. Despite high clinical relevance, gaps in formal training and protocol standardization persist. Enhancing education and harmonizing practices is essential for optimizing LUS integration into acute care.

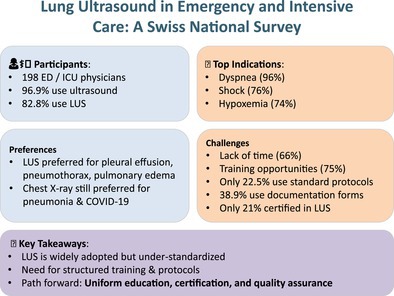

## 1 Introduction

The utilization of lung ultrasound (LUS) has been documented since the early 1990s (Mathis 1992) and has subsequently evolved into a diverse range of applications, ranging from fundamental diagnostic procedures such as the identification of pleural effusions to more sophisticated applications including ultrasound‐guided interventions and the characterization of lung tumors. In recent years, point‐of‐care LUS has gained popularity in emergency and critical care settings, with an increasing number of clinical conditions being diagnosed using this method. These include pneumothorax, pneumonia, and pneumonitis, atelectasis, lung contusion, pulmonary oedema, and interstitial disease. The most evident advantages of LUS over other imaging modalities are the capacity to obtain real‐time images at the bedside, the ability to perform repeated examinations when necessary, the high diagnostic accuracy for various pathologies, and the avoidance of radiation exposure (Lichtenstein et al. 2004; Volpicelli et al. 2012). The international evidence‐based recommendations for LUS are focused on the emergency and critical care settings, emphasizing its importance in the diagnosis and efficient management of such patients (Volpicelli et al. 2012). Indeed, LUS has the potential to improve patient outcomes and decision‐making in the emergency department (ED) and ICU by providing a rapid, accurate, and non‐invasive assessment of lung disease (Ablordeppey et al. 2017; Llamas‐Álvarez et al. 2017; Le Neindre et al. 2023). As a result, it has become an invaluable tool in these settings to guide clinical management and improve patient care (Le Neindre et al. 2023; Xirouchaki et al. 2014), even more so during the COVID‐19 pandemic (Musa et al. 2022).

In recent years, ultrasound has been included in the curriculum of most medical schools in Switzerland, as a certificate of competence in sonography is strongly encouraged to perform and required to bill for ultrasound examinations. The Swiss Society for Ultrasound in Medicine administers and accredits certificates and ultrasound courses. A minimal number of ultrasound examinations (at least 100 or 200, depending on the training component) must be performed under supervision to obtain a certificate of competence.

However, despite the established utility of LUS, there is a paucity of knowledge regarding its current utilization, indications, training requirements, and factors impeding its implementation. Previous studies suggest barriers such as limited training and equipment access may hinder LUS adoption (Zanforlin et al. 2020). Switzerland's decentralized healthcare system and varying ultrasound training programs make it an ideal setting to explore LUS practice heterogeneity. Therefore, we conducted a national online survey to determine the current practice of LUS utilization among emergency and critical care physicians and to provide potentially useful insights for quality improvement initiatives aimed at optimizing the use of LUS in patient care. The objective of this study was to investigate the following aspects of LUS practice: (a) indications for and the frequency of use, (b) level of training, accreditation, and self‐reported skill of practitioners, (c) implementation of standard protocols, (d) preference for ultrasound probes, and (e) barriers to LUS implementation.

## 2 Material and Methods

We conducted an online survey aimed at assessing LUS use, training, protocols, and barriers among emergency and intensive care physicians in Switzerland. The survey was designed following recommendations for survey‐based research (Eysenbach 2004) and was developed with reference to prior studies (Zanforlin et al. 2020) to ensure comprehensiveness and relevance. It consisted of 27 questions (Appendix A), utilizing branching logic to differentiate questions for LUS users and non‐users. Questions were developed in an iterative process involving an expert in point‐of‐care ultrasound (TB). The survey was kept intentionally brief (approximately 7 min to complete) to maintain engagement and minimize respondent fatigue, focusing on the most relevant questions. The questions covered demographics, LUS use frequency, training methods, probe preferences, protocol availability, and barriers, using a mix of multiple‐choice and Likert‐scale questions. The survey was pilot‐tested and refined before dissemination.

The survey was created and administered using the Findmind platform (www.findmind.ch) (Keller 2024). To ensure broad representation across departments, we targeted all 108 EDs and 75 ICUs in Switzerland. Due to the inability to directly access personal contact details of individual physicians within these departments, a snowball sampling approach without limits in sample size was employed. The initial invitations were sent via email to department heads (SGI‐SSMI 2021). Department heads were asked to distribute the survey link to all eligible medical staff, allowing the survey to reach physicians at different experience levels and specialties within their institutions. Snowball sampling was employed due to logistical constraints. However, this approach may introduce selection bias, as motivated or LUS‐interested physicians may be more inclined to respond. Therefore, two reminder emails were sent: the first 15 days after the initial invitation and the second 21 days after that, to encourage maximum participation.

Participation in the survey was voluntary and anonymous. No incentives were provided, and participants were required to provide informed consent at the beginning of the survey.

Ethical committee approval was not required by Swiss regulations, as the study was limited to anonymous data collection from medical professionals and did not include any personal or health‐related patient data.

Data were analyzed using descriptive statistics, with categorical variables presented as frequencies and percentages, and comparisons between groups conducted using chi‐squared or Mann–Whitney U‐tests for nonparametric data. We performed all analyses in Microsoft Excel and STATA 14 (StataCorp, College Station, TX, USA).

## 3 Results

**FIGURE 1:**
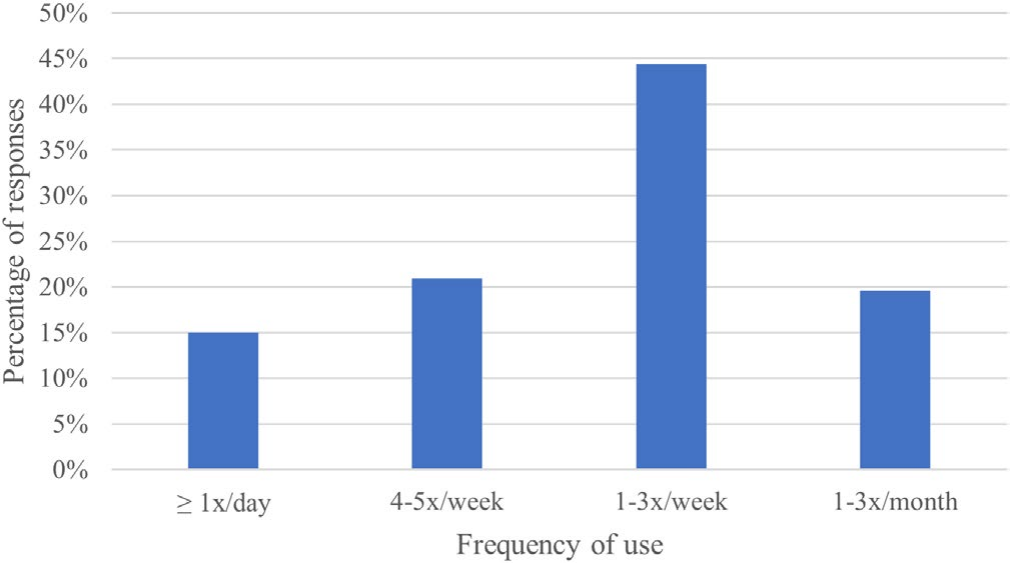
LUS‐users with frequency of use.

The survey was answered by 198 participants from across Switzerland, with 186 completing all survey items. Due to the snowball sampling approach, the exact number of invited physicians could not be determined, precluding response rate calculation. Demographic details are provided in Table 1, showing a balanced distribution across age, experience, and hospital type. Of the participants, 49.0% were emergency department (ED) physicians, 42.3% were intensive care unit (ICU) physicians, and 8.7% represented other specialties. A substantial majority (96.9%, *n* = 190) reported using ultrasound in their clinical practice, with 80.5% (*n* = 153) using LUS at least monthly. Among LUS users, 44.4% used LUS one to three times per week (Figure 1).

**TABLE 1:** Demographic characteristics of participants.

Characteristics	*n* (%)
Age	
< 30 years	32 (16.2%)
31–40 years	66 (33.3%)
41–50 years	47 (23.7%)
51–60 years	44 (22.2%)
> 60 years	9 (4.5%)
Gender	
Male	115 (58.1%)
Female	82 (41.4%)
Other	1 (0.5%)
Country of medical examination	
Switzerland	146 (73.7%)
Germany	22 (11.1%)
Italy	12 (6.1%)
France	2 (1.0%)
Other European country	14 (7.1%)
Non‐European country	2 (1.0%)
Years of experience as a physician	
< 5 years	39 (19.7%)
5–10 years	38 (19.2%)
11–20 years	64 (32.3%)
21–30 years	38 (19.2%)
> 30 years	19 (9.6%)
Place of work	
University Hospital	39 (19.7%)
Cantonal Hospital	75 (37.9%)
Regional Hospital	62 (31.3%)
Private clinic	17 (8.6%)
Rehabilitation clinic	2 (1.0%)
Other	3 (1.5%)
Medical field	
Emergency medicine	96 (49.0%)
Intensive care medicine	83 (42.3%)
Other	17 (8.7%)
Region	
Lake Geneva region	36 (18.4%)
Espace Mittelland	55 (28.1%)
Northwestern Switzerland	24 (12.2%)
Zurich region	32 (16.3%)
Eastern Switzerland	13 (6.6%)
Central Switzerland	17 (8.7%)
Ticino	19 (9.7%)
Ultrasound use	
Ultrasound users	190 (96.9%)
Ultrasound non‐users	6 (3.1%)
Lung ultrasound use (at least once a month)	
Lung ultrasound users	153 (81.4%)
Lung ultrasound non‐users	35 (18.6%)

### 3.1 LUS‐Users

**FIGURE 2:**
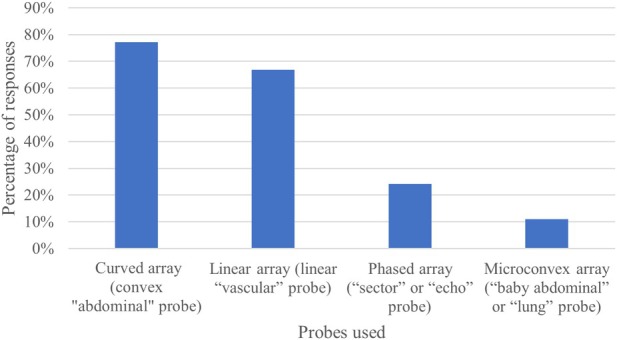
Probes used to perform LUS.

**FIGURE 3:**
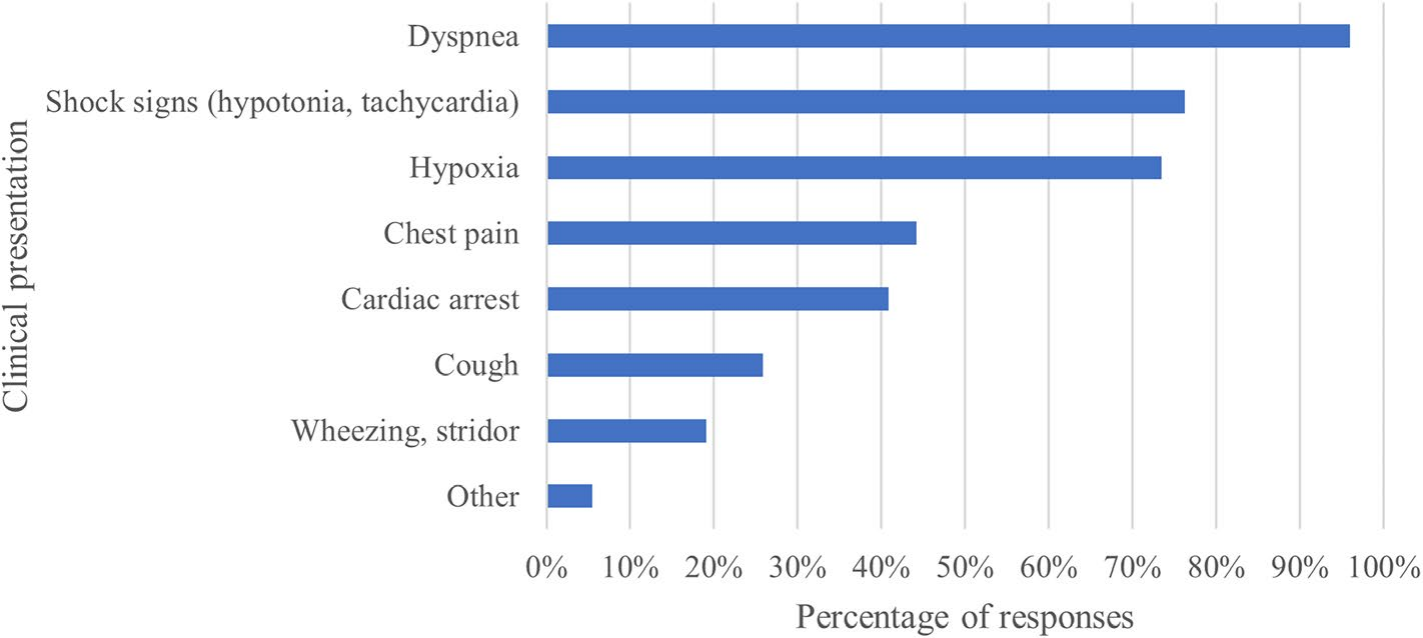
Main indications for performing LUS.

LUS was most commonly performed with curved (77.2%) and linear (66.9%) probes, while phased array (24.1%) and microconvex (11.0%) were less frequently used (Figure 2). LUS was rated highly relevant in clinical practice, with a median relevance score of 8 (on a 1–10 scale). The primary indications for LUS included dyspnea (95.9%), shock (76.2%), and hypoxemia (73.5%) (Figure 3). LUS was the preferred imaging modality for pleural effusion (91.8%), pneumothorax (82.3%), and acute pulmonary edema (53.1%), whereas for conditions like pneumonia, pulmonary consolidation, and COVID‐19, the majority of responders still preferred chest X‐rays. For procedural guidance, LUS was commonly used in thoracentesis (97.2%) and, less frequently, for needle aspiration of pneumothorax (37.2%) and chest tube insertion (38.6%). The availability of standardized LUS protocols and documentation forms was reported by 22.5% and 38.9% of respondents, respectively.

**FIGURE 4:**
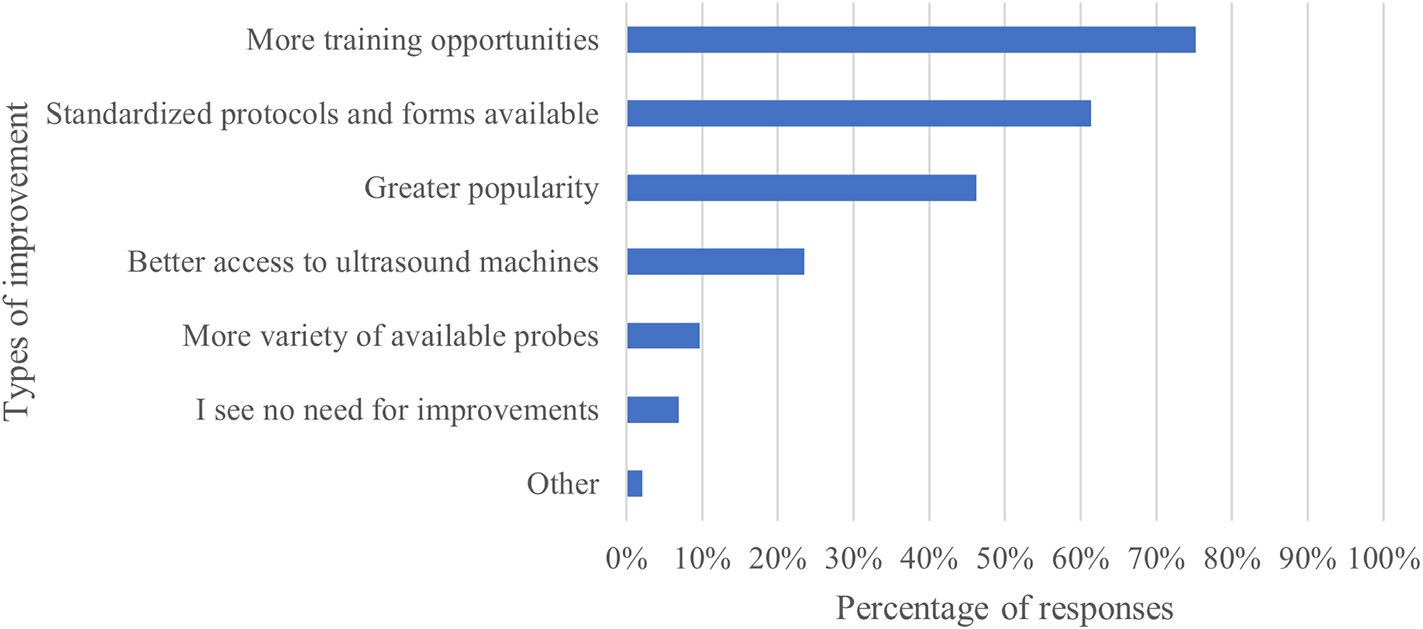
Opportunities for improvement regarding LUS.

In terms of training, most LUS users had developed their skills through peer mentorship (74.3%), self‐learning (65.8%), general ultrasound courses (55.9%), and specific chest or LUS courses (48.0%). We found no significant differences in the type of LUS training between ICU and non‐ICU physicians. Only 21.1% held a formal certification in lung or thoracic ultrasound, predominantly issued by the Swiss Society of Ultrasound in Medicine. Skill levels were self‐rated as average (38.6%) or fairly good (33.3%), with fewer participants rating their skills as basic (17.6%) or very good (10.5%). The primary suggestions for improving LUS use included offering more training opportunities (75.2%) and implementing standardized protocols and documentation (61.4%) (Figure 4).

### 3.2 LUS Utilization

Physicians with more than 10 years of experience reported using LUS more frequently than those with less than 10 years of experience (*p* = 0.008) (Table 2), reflecting greater familiarity and comfort with the modality. The more experienced group also had a significantly higher rate of formal LUS training, including certification (27.6% vs. 9.3%, *p* = 0.008), whereas the less experienced group more often relied on mentoring as their primary mode of learning. ICU physicians were more likely to use LUS for invasive procedures, such as needle aspiration of pneumothorax (46.3% vs. 29.5%, *p* = 0.037) and chest tube placement (52.2% vs. 26.9%, *p* = 0.002), reflecting the typical procedures in intensive care (Table 3). In terms of equipment, ICU physicians more frequently used phased array (32.8% vs. 16.7%, *p* = 0.023) and microconvex probes (17.9% vs. 5.1%, *p* = 0.014) compared to non‐ICU physicians, potentially reflecting differences in diagnostic focus and available resources within these settings. Both groups shared similar views on the importance of further LUS training, improved access to ultrasound devices, and the need for standardized protocols and documentation forms.

**TABLE 2:** Comparison of LUS‐users with more versus less than 10 years of clinical experience.

Variable	Total *n*	Clinical experience < 10 years	Clinical experience > 10 years	*p*
Type of US used	153			
Abdominal US	121	43 (79.6)	78 (78.8)	0.903
Vascular US	111	31 (57.4)	80 (80.8)	0.002
E‐FAST	124	48 (88.9)	76 (76.8)	0.067
Echocardiography	104	31 (57.4)	73 (73.7)	0.039
LUS	151	52 (96.3)	99 (100.0)	0.054
Frequency of LUS‐use	153			0.008
≥ 1×/day	23	3 (5.6)	20 (20.2)	
4–5×/week	32	7 (13.0)	25 (25.3)	
1–3×/week	68	32 (59.3)	36 (36.4)	
1–3×/month	30	12 (22.2)	18 (18.2)	
Assessment of LUS‐skills	153			< 0.001
Very good skills	16	3 (5.6)	13 (13.1)	
Fairly good skills	51	10 (18.5)	41 (41.4)	
Average skills	59	22 (40.7)	37 (37.4)	
Basic skills	27	19 (35.2)	8 (8.1)	
LUS training	152			
General US course	85	31 (57.4)	54 (55.1)	0.784
Specific LUS course	73	17 (31.5)	56 (57.1)	0.003
Self‐learning	100	31 (57.4)	69 (70.4)	0.106
Mentor	113	46 (85.2)	67 (68.4)	0.023
LUS certificate	32	5 (9.3)	27 (27.6)	0.008
Indications for performing LUS	147			
Chest pain	65	23 (44.2)	42 (44.2)	0.998
Dyspnea	141	51 (98.1)	90 (94.7)	0.328
Cough	38	11 (21.2)	27 (28.4)	0.336
Wheezing	28	11 (21.2)	17 (17.9)	0.630
Hypoxia	108	37 (71.2)	71 (74.7)	0.638
Shock	112	36 (69.2)	76 (80.0)	0.143
Cardiac arrest	60	20 (38.5)	40 (42.1)	0.667
LUS for interventions	145			
Needle aspiration of pneumothorax	54	16 (30.8)	38 (40.9)	0.228
Thoracentesis	141	50 (96.2)	91 (97.8)	0.550
Chest tube insertion	56	10 (19.2)	46 (49.5)	< 0.001
LUS preferred over chest X‐ray for	146			
Pleural effusion	135	48 (92.3)	87 (92.6)	0.957
Pneumothorax	121	44 (84.6)	77 (81.9)	0.678
Pneumonia	26	2 (3.8)	24 (25.5)	0.001
COVID‐19	26	3 (5.8)	23 (24.5)	0.005
Consolidation	36	9 (17.3)	27 (28.7)	0.125
Pulmonary edema	78	28 (53.8)	50 (53.2)	0.939
ARDS	22	5 (9.6)	17 (18.1)	0.171
Interstitial syndrome	31	8 (15.4)	23 (24.5)	0.199
Lung contusion	25	6 (11.5)	19 (20.2)	0.183
US probe used for LUS	145			
Curved array	112	44 (84.6)	68 (73.1)	0.113
Linear array	97	30 (57.7)	67 (72.0)	0.078
Phased array	35	8 (15.4)	27 (29.0)	0.065
Microconvex array	16	7 (13.5)	9 (9.7)	0.485

Abbreviations: ICU, intensive care unit; LUS, lung ultrasound; US, ultrasound.

**TABLE 3:** Comparison of LUS‐users working in ICU versus non‐ICU.

Variable	Total *n*	ICU physicians	Non‐ICU physicians	*p*
Type of US used	153			
Abdominal US	121	45 (63.4)	76 (92.7)	< 0.001
Vascular US	111	60 (84.5)	51 (62.2)	0.002
E‐FAST	124	45 (63.4)	79 (96.3)	< 0.001
Echocardiography	104	60 (84.5)	44 (53.7)	< 0.001
LUS	151	71 (100.0)	80 (97.6)	0.185
Frequency of LUS‐use	153			0.956
≥ 1×/day	23	10 (14.1)	13 (15.9)	
4–5×/week	32	15 (21.1)	17 (20.7)	
1–3×/week	68	33 (46.5)	35 (42.7)	
1–3×/month	30	13 (18.3)	17 (20.7)	
LUS‐skills	153			0.041
Very good skills	16	6 (8.5)	10 (12.2)	
Fairly good skills	51	32 (45.1)	19 (23.2)	
Average skills	59	23 (32.4)	36 (43.9)	
Basic skills	27	10 (14.1)	17 (20.7)	
LUS training	152			
General US course	85	39 (54.9)	46 (56.8)	0.818
Specific LUS course	73	39 (54.9)	34 (42.0)	0.111
Self‐learning	100	47 (66.2)	53 (65.4)	0.921
Mentor	113	53 (74.6)	60 (74.1)	0.936
LUS certificate	32	17 (23.9)	15 (18.5)	0.413
Standardized protocol for performing LUS available	151			0.512
Yes	34	17 (24.3)	17 (21.0)	
No	92	44 (62.9)	48 (59.3)	
I don't know	25	9 (12.9)	16 (19.8)	
Standardized form for documenting LUS available	149			0.628
Yes	58	25 (36.8)	33 (40.7)	
No	79	36 (52.9)	43 (53.1)	
I don't know	12	7 (10.3)	5 (6.2)	
Indications for performing LUS	147			
Chest pain	65	18 (26.9)	47 (58.8)	< 0.001
Dyspnea	141	65 (97.0)	76 (95.0)	0.539
Cough	38	17 (25.4)	21 (26.2)	0.904
Wheezing	28	10 (14.9)	18 (22.5)	0.244
Hypoxia	108	56 (83.6)	52 (65.0)	0.011
Shock	112	48 (71.6)	64 (80.0)	0.236
Cardiac arrest	60	29 (43.3)	31 (38.8)	0.578
LUS for interventions	145			
Needle aspiration of pneumothorax	54	31 (46.3)	23 (29.5)	0.037
Thoracentesis	141	65 (97.0)	76 (97.4)	0.877
Chest tube insertion	56	35 (52.2)	21 (26.9)	0.002
LUS preferred over chest X‐ray for	146			
Pleural effusion	135	65 (97.0)	70 (88.6)	0.055
Pneumothorax	121	52 (77.6)	69 (87.3)	0.120
Pneumonia	26	11 (16.4)	15 (19.0)	0.686
COVID‐19	26	13 (19.4)	13 (16.5)	0.643
Consolidation	36	22 (32.8)	14 (17.7)	0.035
Pulmonary edema	78	36 (53.7)	42 (53.2)	0.945
ARDS	22	8 (11.9)	14 (17.7)	0.331
Interstitial syndrome	31	13 (19.4)	18 (22.8)	0.619
Lung contusion	25	6 (9.0)	19 (24.1)	0.016
US probe used for LUS	145			
Curved array	112	51 (76.1)	61 (78.2)	0.765
Linear array	97	45 (67.2)	52 (66.7)	0.949
Phased array	35	22 (32.8)	13 (16.7)	0.023
Microconvex array	16	12 (17.9)	4 (5.1)	0.014

Abbreviations: ICU, intensive care unit; LUS, lung ultrasound; US, ultrasound.

### 3.3 LUS Indications

More experienced physicians were more likely to use LUS for indications such as chest tube insertion, pneumonia, and COVID‐19 diagnosis (Table 2). ICU physicians more frequently used LUS for patients with hypoxia, while non‐ICU physicians reported using it more often for assessing chest pain. ICU physicians also demonstrated a greater preference for LUS over chest radiography in diagnosing conditions like pleural effusion and consolidation (Table 3).

### 3.4 LUS Non‐Users

Thirty‐five respondents (18.6%) did not regularly use LUS, primarily due to time constraints for training (65.9%) or because other physicians in the department performed LUS (29.3%). A majority (90.2%) expressed interest in LUS training, with a preference for blended learning (56.8%) and one‐day courses (43.2%).

## 4 Discussion

This nationwide survey provides a preliminary overview of LUS practices, training, and barriers among emergency and intensive care physicians in Switzerland. We found that 82.8% of responders incorporate LUS into their clinical practice, emphasizing its critical role in assessing acute conditions. LUS is predominantly used for the assessment of patients with dyspnea, shock, and hypoxemia, as well as for guiding thoracentesis. It is the preferred imaging modality for evaluating pleural effusion, pneumothorax, and acute pulmonary edema.

Our comparison between ICU and non‐ICU physicians revealed distinct usage patterns in ultrasound modalities, with notable differences emerging based on clinical experience: more experienced physicians reported higher LUS utilization, more advanced training, and a greater likelihood of holding LUS certification. Notably, only 21% of LUS users in our survey were certified in pulmonary or thoracic ultrasound, although certified providers are not necessarily more competent or better trained than non‐certified ones.

### 4.1 Relationship to Previous Research

Our results are consistent with previous work on the use of LUS in other countries (Zanforlin et al. 2020; Berlet et al. 2014; Calamai et al. 2017). A survey of Italian pulmonologists by Zanforlin et al. (2020) showed that LUS is widely used and considered a clinically relevant tool. Consistent with our findings, LUS was mainly used for both diagnostic and interventional procedures and for clinical conditions such as pleural effusion, pneumothorax, pneumonia, heart failure, and dyspnea. The percentage of respondents who did not use LUS (21%) was similar to ours (18.6%). However, unlike in Switzerland, the main obstacle in Italy seems to be the availability of an ultrasound machine, with other reasons being lack of protected time and training and use of the technique by other specialists. Calamai et al. (2017) surveyed 87 Italian ICUs and found that LUS was performed in 94.3% of responding units to evaluate known or suspected pleural effusion, pneumothorax, atelectasis, congestion, and consolidation. As in our survey, standardized reporting forms were rarely available (only 11% of ICUs) and the ultrasound probes used were convex (95.7%), linear (97.1%), phased array (81.7%), and microconvex (7%). Finally, in a survey of accredited sonographers in Germany, most respondents used linear or curvilinear probes, while microconvex probes were used the least (Berlet et al. 2014).

### 4.2 Implications and Future Directions

Our survey indicates that LUS is the preferred imaging modality specifically for pleural effusion, pneumothorax, and acute pulmonary edema. For other conditions, however, most clinicians continue to favor chest X‐ray, despite evidence from several studies demonstrating LUS's superior diagnostic accuracy for detecting lung consolidation, atelectasis, pneumonia, and lung contusion (Lichtenstein et al. 2004; Shrestha et al. 2018; Alrajab et al. 2013; Mateos Gonzalez et al. 2021; Xia et al. 2016; Hosseini et al. 2015). Additionally, while microconvex probes are recommended as the optimal choice by some experts (Lichtenstein 2014), they are seldom used in practice. This may be due to the limited availability of such probes or the practicality that curvilinear and linear probes, which are more commonly accessible, can often provide the necessary LUS views and measurements. In line with recent expert recommendations from the ESICM (Robba et al. 2021), which emphasize standardizing critical care ultrasonography skills for intensivists, our results further reinforce the call for accessible, more structured training pathways, aligned with professional recommendations, to optimize LUS proficiency in emergency and intensive care settings. This is especially important considering that greater ultrasound proficiency increases the possibility of identifying unsuspected and potentially dangerous structural abnormalities and may, therefore, have a direct impact on patient outcomes (Corvino et al. 2024).

### 4.3 Strengths and Limitations

This study represents the first national survey of LUS practices among emergency and critical care physicians in Switzerland, providing valuable insights into LUS usage, training, and barriers to its broader implementation. By capturing data from a diverse sample of emergency and ICU departments across Switzerland, we gained an overview of current practices and identified key areas for improvement, such as the need for standardized protocols and enhanced training opportunities. The findings have important implications for guiding national initiatives aimed at promoting more uniform LUS usage and quality assurance in acute care settings.

However, this study has several limitations, primarily due to the use of a snowball sampling method. While this approach enabled broad and efficient survey dissemination, it also introduced selection bias, as participants were recruited indirectly through department heads. This may have resulted in an over‐representation of those already engaged or interested in LUS, which could skew the findings. Additionally, since we could not track how many individuals received or forwarded the survey, it was impossible to calculate an accurate response rate, limiting the study's external validity. This limitation means that our findings cannot be assumed to represent the full spectrum of emergency and ICU physicians in Switzerland, which may impact the generalizability of the results. Of 198 participants, 12 had incomplete responses. Analyses used listwise deletion, with minimal impact on results due to the low missing data rate (6%). We used a relatively short questionnaire that relied on categorical responses. Although more detailed information might have been useful, we believe that a more time‐consuming survey would have resulted in a lower response rate. Finally, the questionnaire was only available in English. This may have discouraged participants with limited English proficiency from responding. However, most physicians in Switzerland are proficient in English, and we do not believe that providing the survey in other languages would have significantly altered our results.

## 5 Conclusions

This nationwide survey provides exploratory insights into the current use and preferences of LUS among emergency and critical care physicians in Switzerland, revealing both its widespread adoption for specific acute conditions and areas where practice gaps remain. The observed variability in LUS use, training, and certification highlights the need for more structured, accessible training programs and the establishment of standardized protocols to ensure consistency and accuracy in LUS applications in clinical practice.

## Ethics Statement

Ethical committee approval was not required by Swiss regulations, as the study was limited to anonymous data collection from medical professionals and did not include any personal or health‐related patient data.

## Consent

The authors have nothing to report.

## Conflicts of Interest

The authors declare no conflicts of interest relevant to this work. Unrelated to this work, L.C. has received research grants from the Research Council of the Cantonal Hospital Aarau, educational and travel grants from Hamilton Medical, educational grants from Fresenius Medical Care, speaker honoraria from OrphaSwiss, and is an advisory board member for OrphaSwiss.

## Data Availability

The data that support the findings of this study are available from the corresponding author upon reasonable request.

## Appendix A

See Appendix A

Detailed Survey Questions and Response Options

1. Informed consent: By clicking “continue” you acknowledge that you have read and understand all the above information. You agree to participate in this survey and to the analysis of the data.
  - Continue
2. Your age:
  - < 30 years
  - 31–40 years
  - 41–50 years
  - 51–60 years
  - > 60 years
3. Your gender:
  - Male
  - Female
  - Other (please specify)
4. Where did you take your final medical examination?
  - Switzerland
  - Germany
  - Italy
  - France
  - Other European country
  - Non‐European country
5. How many years have you been working clinically as a physician?
  - < 5 years
  - 5–10 years
  - 11–20 years
  - 21–30 years
  - > 30 years
6. What type of hospital do you work in?
  - University Hospital
  - Cantonal Hospital
  - Regional Hospital
  - Private clinic
  - Rehabilitation clinic
  - Other (please specify)
7. In which field do you work predominantly?
  - Emergency Medicine
  - Intensive Care Medicine
  - Other (please specify)
8. In which part of the country do you work?
  - Lake Geneva region (Vaud, Valais, Geneva)
  - Espace Mittelland (Bern, Fribourg, Solothurn, Neuchâtel, Jura)
  - Northwestern Switzerland (Basel, Aargau)
  - Zurich Region
  - Eastern Switzerland (Glarus, Schaffhausen, Appenzell, St. Gallen, Graubünden, Thurgau)
  - Central Switzerland (Luzern, Uri, Schwyz, Obwalden, Nidwalden, Zug)
  - Ticino
9. Do you use ANY TYPE of ultrasound in your clinical practice?
  - Yes (→ *question 10*)
  - No (→ *question 25*)
10. What type of ultrasound exam do you perform? (Tick all that apply)
  - Abdominal ultrasound
  - Vascular ultrasound
  - E‐FAST
  - Echocardiography or cardiac sonography
  - Thoracic or lung ultrasound
  - Other (please specify)
11. Do you use LUNG ultrasound regularly (at least once a month)?
  - Yes (→ *question 12*)
  - No (→ *question 25*)
12. How often do you use LUNG ultrasound in your clinical practice?
  - ≥ 1×/day
  - 4–5×/week
  - 1–3×/week
  - 1–3×/month
13. How would you rate your skills in using LUNG ultrasound?
  - Very good skills
  - Fairly good skills
  - Average skills
  - Basic skills
14. How did you learn LUNG ultrasound? (Tick all that apply)
  - General ultrasound course
  - Specific ultrasound course (chest, lung)
  - Self‐learning (books, E‐learning, videos)
  - Mentoring/Coaching by other physicians
15. Do you have a certificate/diploma in LUNG or THORACIC ultrasound?
  - Yes (→ *question 16*)
  - No (→ *question 17*)
16. From which association/society?
  - Free text field
17. Does your institution have standardized protocols for performing LUNG ultrasound examinations?
  - Yes
  - No
  - I do not know
18. Does your institution have standardized forms/templates for documenting LUNG ultrasound findings?
  - Yes
  - No
  - I do not know
19. How do you rate the clinical relevance of LUNG ultrasound for your daily practice? (On a scale from 1 to 10 (1 = completely irrelevant; 10 = extremely relevant))
  - Scale 1–10
20. Which are the main indications for you to perform LUNG ultrasound? (Tick all that apply)
  - Chest pain
  - Dyspnea
  - Cough
  - Wheezing, stridor
  - Hypoxia
  - Shock signs (hypotonia, tachycardia)
  - Cardiac arrest
  - Other (please specify)
21. For which of the following conditions do you prefer LUNG ultrasound over chest X‐ray as the diagnostic modality of choice? (Tick all that apply)
  - Pleural effusion
  - Pneumothorax
  - Pneumonia
  - COVID‐19
  - Lung consolidation
  - Acute pulmonary edema
  - ARDS
  - Interstitial syndrome
  - Lung contusion
  - Other (please specify)
22. Which probe do you use for LUNG ultrasound? (Tick all that apply)
  - Curved array (convex “abdominal” probe)
  - Linear array (linear “vascular” probe)
  - Phased array (“sector” or “echo” probe)
  - Microconvex array (“baby abdominal” or “lung” probe)
23. For which thoracic interventions do you use LUNG ultrasound? (Tick all that apply)
  - Needle aspiration of pneumothorax
  - Thoracentesis of fluid
  - Chest tube insertion
  - Other (please specify)
24. Where would you like to see improvements regarding LUNG ultrasound? (Tick all that apply)
  - Greater popularity
  - More training opportunities
  - Better access to ultrasound machines
  - More variety of available probes
  - Standardized protocols and forms available in the institution
  - I see no need for improvements
  - Other (please specify)
25. Why don't you perform LUNG ultrasound? (Tick all that apply)
  - No need
  - No time for training or self‐study
  - No ultrasound machine available or not allowed to use it
  - No suitable ultrasound probe available
  - Lung ultrasound is performed by other physicians
  - Other (please specify)
26. Would you be interested in taking a LUNG ultrasound course?
  - Yes (→ *question 27*)
  - No (→ *finished survey*)
27. What kind of course would you prefer?
  - Hands‐on training
  - Online course/E‐learning
  - Blended learning (combination of hands‐on training and online course)
28. What is the maximum amount of time you would be willing to invest in a training course?
  - 0.5 days
  - 1 day
  - 2 days
  - 3 days
